# Role of Electrical Activity in Horizontal Axon Growth in the Developing Cortex: A Time-Lapse Study Using Optogenetic Stimulation

**DOI:** 10.1371/journal.pone.0082954

**Published:** 2013-12-23

**Authors:** Olga Malyshevskaya, Yoshihiro Shiraishi, Fumitaka Kimura, Nobuhiko Yamamoto

**Affiliations:** 1 Laboratory of Cellular and Molecular Neurobiology, Graduate School of Frontier Biosciences, Osaka University, Suita, Osaka, Japan; 2 Department of Molecular Neuroscience, Graduate School of Medicine, Osaka University, Suita, Osaka, Japan; Osaka University Graduate School of Medicine, Japan

## Abstract

During development, layer 2/3 neurons in the neocortex extend their axons horizontally, within the same layers, and stop growing at appropriate locations to form branches and synaptic connections. Firing and synaptic activity are thought to be involved in this process, but how neuronal activity regulates axonal growth is not clear. Here, we studied axonal growth of layer 2/3 neurons by exciting cell bodies or axonal processes in organotypic slice cultures of the rat cortex. For neuronal stimulation and morphological observation, plasmids encoding channelrhodopsin-2 (ChR2) and DsRed were coelectroporated into a small number of layer 2/3 cells. Firing activity induced by photostimulation (475 nm) was confirmed by whole-cell patch recording. Axonal growth was observed by time-lapse confocal microscopy, using a different excitation wavelength (560 nm), at 10–20-min intervals for several hours. During the first week *in vitro*, when spontaneous neuronal activity is low, DsRed- and ChR2-expressing axons grew at a constant rate. When high-frequency photostimulation (4 or 10 Hz) for 1 min was applied to the soma or axon, most axons paused in their growth. In contrast, lower-frequency stimulation did not elicit this pause behavior. Moreover, in the presence of tetrodotoxin, even high-frequency stimulation did not cause axonal growth to pause. These results indicate that increasing firing activity during development suppresses axon growth, suggesting the importance of neuronal activity for the formation of horizontal connections.

## Introduction

Neocortical circuits are known to be established not only by genetic mechanisms but also by environmental factors such as sensory-evoked neuronal activity. Among the best examples of this formation process are the projections from the thalamic nuclei to the cortices [1]. Intrinsic cortical circuits are also thought to be established based on genetic and activity-dependent mechanisms. Axon collaterals from layer 2/3 pyramidal neurons, called horizontal axons, connect cells within the same layer, and these connections provide a substrate for lateral interactions across cortical columns [2]–[8]. In the visual cortex of higher mammals, the horizontal connections primarily link cortical cells in columns having the same orientation [9]–[15]. Horizontal connections have also been found in other cortical areas, and are the basis of information processing in the neocortex [16]–[23].

During development, horizontal axons start to grow in all directions from the cell body, and then display further elongation and initial branch formation at later periods [24], [25]. Ultimately, only specific locations undergo robust and complex branch formation, whereas other regions show selective retraction of inappropriate connections. This developmental scheme is similar among different mammalian species, albeit with some differences in the length of maturation period [24]–[28].

Neuronal activity is essential for the formation of horizontal connections, since the organization of these connections is disrupted by sensory deprivation and by blockade of action potentials [29]–[32]. Furthermore, our previous *in vitro* studies have demonstrated that firing and synaptic activity promote horizontal axon branching [33], [34]. Nevertheless, how electrical activity contributes to the regulation of horizontal axon growth remains unknown.

To address this issue, here we studied axon growth of layer 2/3 cells in organotypic cortical slice culture by manipulating firing activity using an optogenetic technique [35]–[38]. Light-gated algal channel channelrhodopsin-2 (ChR2) enables neuronal activity to be generated in a cell-specific way with high temporal resolution and is therefore an excellent tool to study the complex mechanism of cortical network formation. The results revealed that horizontally elongating axons pause in their growth after high-frequency stimulation, suggesting that developing firing activity contributes to circuit formation by regulating axonal growth.

## Materials and Methods

### Animals and Ethics Statement

Sprague-Dawley rats were used for this study. All experiments were performed according to guidelines laid down by the animal welfare committees of Osaka University and the Japanese Neuroscience Society. The protocol was approved by the Committee on the Ethics of Animal Experiments of Osaka University (Permit Number: FBS 07–037).

### Organotypic Slice Culture

To observe horizontal axons, we used a previously described organotypic slice culture system [39], [40]. In this culture, cortical circuits including horizontal connections are reproduced with the cortical laminar structure preserved [33]. In brief, the occipital region of the cortex was dissected from postnatal day 0 (P0) or P1 rats. The slices were placed on a membrane filter (MilliCell-CM Low Height PICMORG-50, Millipore, Billerica, MA) coated with rat tail collagen. The culture medium consisted of a 1∶1 mixture of Dulbecco’s modified essential medium and Ham’s F12 (Life Technologies, Carlsbad, CA) with several supplements including insulin and transferrin [40]. The cultures were maintained at 37°C in an environment of humidified 95% air and 5% CO_2_.

### Plasmids

A plasmid containing hChR2(H134R)-EYFP was obtained from Dr. Karl Deisseroth, Stanford University [41], and the coding region was placed in pCAGGS vector (a generous gift from Dr. Naofumi Uesaka, University of Tokyo) [42]. The plasmid was purified using a MaxiPrep kit (PureLink, Life Technologies, Carlsbad, CA) according to the supplier’s protocol, and dissolved in Hanks’ solution at appropriate concentrations. pCAGGS-DsRed and pCAGGS-EYFP plasmids were prepared in a similar way.

### Local Cell Electroporation

Local electroporation with glass microelectrodes was performed after 1–2 days in culture [33]. In brief, pCAGGS-DsRed (2–3 µg/µl) or a mixture of pCAGGS-hChR2 (H134R)-EYFP (3 µg/µl) and pCAGGS-DsRed (2 µg/µl) was pressure-ejected with a glass micro capillary (70-µm diameter) onto the surface of the slice. In some cases, pCAGGS-EYFP (2 µg/µl) was used. Electrical pulses (10 trains of 200 square pulses of 1-msec duration, 200 Hz, 400–500 µA) were then applied with another glass microelectrode (200-µm diameter).

### Morphological Time-lapse Observation of Growing Axons

At 3–10 days *in vitro* (DIV), a culture dish containing cortical slices was sealed with a coverslip and transferred to a microscope stage. During the 4–5-h observation, the slices were maintained at 37°C using a heating apparatus (Kokensha Engineering, Tokyo, Japan) and supplied with a 95% air and 5% CO_2_ humidified gas mixture [43]. The objective lens was coiled with a band-heater (Thermoplate, Tokai Hit, Shizuoka, Japan) to prevent condensation.

Morphological observation was performed using a confocal laser scanning microscope (Nikon C2, Nikon Instech, Tokyo, Japan) with a 10x objective lens (Nikon Plan Fluor, NA, 0.3) at 560-nm excitation (solid-state laser). After the cultures had been incubated for at least 1 h, images were acquired at 10–20-min intervals with 3–5-µm steps (3–4 optical sections). The observed axons were mostly traced back to cell bodies. Axons were followed for at least 30 min before photostimulation (see below). In both control and experimental conditions, 560-nm wavelength excitation light was used to observe growth cone behavior.

### Photostimulation

A solid-state illuminator (475 nm peak wavelength; maximal power: 20 mW; Lumencor SPECTRA, Lumencor, Beaverton, OR) was used for photostimulation through the objective lens. The beam position was controlled manually through the microscope stage. The intensity of the light stimulus falling into the beam-focused area of a cortical slice was adjusted using a Lumencor Remote Control Accessory and measured with a light power meter (Laser Scan, Coherent, Santa Clara, CA). The duration and frequency of the light stimulus were controlled by applying square electrical pulses from a pulse generator (Master-8, AMPI, Jerusalem, Israel).

For time-lapse morphological experiments, blue light with the following pulse frequencies and duration was delivered: 0.1 Hz (duration: 200 msec, 60 pulses), 1 Hz (1 msec, 60 pulses), 4 Hz (200 msec, 240 pulses), and 10 Hz (1 msec, 600 pulses). After photostimulation, cortical cultures were observed for 1–2 h. At the end of the experiment, to verify that cells or axons were expressing sufficient levels of CAGGS-hChR2(H134R)-EYFP, blue light (475 nm) was applied to the slice and emitted fluorescence was detected.

Axonal growth was observed by applying light stimuli with different intensities to DsRed/ChR2-EYFP-cotransfected cells. We confirmed that a light intensity of 1.4 mW/mm^2^ was efficient in producing action potentials in the transfected cells (see below) without causing any photodynamic damage.

### Patch-clamp Experiments

To confirm that the photostimulation induced action potentials in ChR2-expressing cells, whole-cell patch-clamp recording was carried out in slice cultures at room temperature. ChR2-EYFP-expressing cells were identified using an epifluorescence microscope (BX51W1, Olympus, Tokyo, Japan) equipped with a 40x water immersion objective (Olympus, NA, 0.8). The excitation light beam was introduced through the objective lens. The minimal light intensity for ChR2 excitation was determined by applying blue light of various intensities.

Resistance of patch pipettes was 7–8 MΩ when they were filled with an intracellular solution containing (in mM) 130 potassium methanesulfonate (CH_3_SO_3_K), 10 HEPES (C_8_H_18_N_2_O_4_S), 10 potassium chloride (KCl), 0.5 EGTA (C_14_H_24_N_2_O_10_), 0.1 spermine (C_10_H_26_N_4_), and 10 phosphocreatine (C_4_H_10_N_3_O_5_P) (pH 7.3, adjusted with KOH, 285–290 mOsm). The composition of standard bathing solution was (in mM) 124 sodium chloride (NaCl), 3 potassium chloride (KCl), 2 calcium chloride (CaCl_2_), 1.3 magnesium sulfate (MgSO_4_), 1.2 monopotassium phosphate (KH_2_PO_4_), 26 sodium hydrogen carbonate (NaHCO_3_), and 10 glucose (pH 7.4). The solution was bubbled with 95% O_2_ and 5% CO_2_.

The membrane voltage was recorded with a Multiclamp 700 A amplifier (Molecular Devices, Sunnyvale, CA), low-pass filtered at 5 kHZ, digitally sampled at 10–20 kHz, and monitored with pCLAMP 9.0 (Molecular Devices) and analyzed off-line using either Clampfit 9.0 or AxoScope 10.2 software.

### Pharmacological Drug Application

To suppress firing activity, 1 µM TTX (Seikagaku-Kogyo, Tokyo, Japan) was added 1 h before the time-lapse experiment.

### Image Processing and Statistical Analysis

Axon growth in the time-lapse study was measured using image processing software (ImageJ 1.45 s). Values are presented with ± standard error of the mean (SEM). Quantitative analysis (Student’s *t*-test) was performed using Microsoft Excel software.

## Results

### Axon Growth of Layer 2/3 Neurons in Organotypic Slice Cultures

Axonal growth of cortical cells was studied using an organotypic slice culture technique, which allowed us to perform gene manipulation and subsequent time-lapse observation easily. Furthermore, we could assess axon behavior in a cellular environment which is close to that *in vivo*, as cortical cell arrangement such as laminar structure is well preserved in this culture system [40].

**Figure 1:**
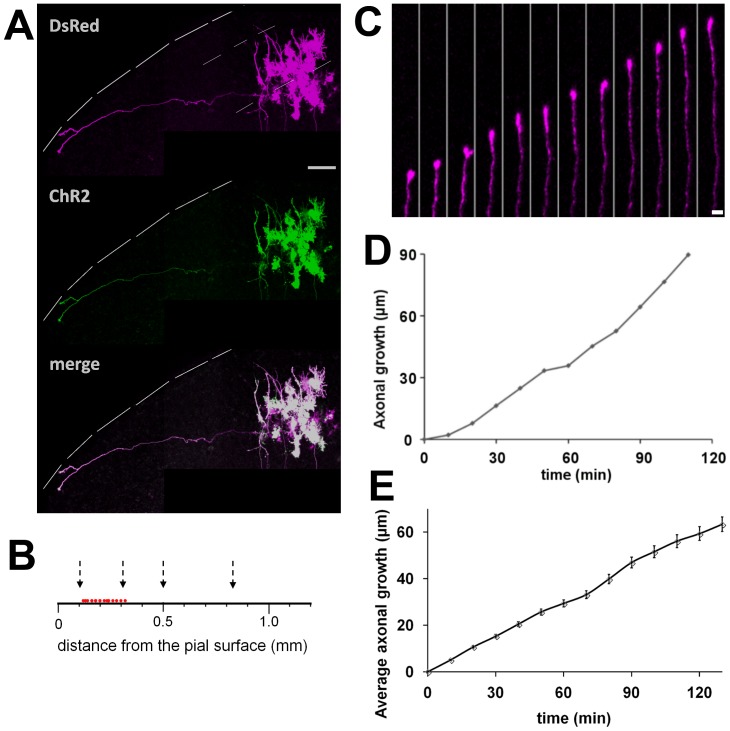
Horizontal axon growth in cortical slice culture. **A,** DsRed and ChR2-EYFP plasmids were introduced into several layer 2/3 cells at 1 DIV, and labeled axons were observed by confocal microscopy (560 nm excitation) at 4 DIV. This excitation does not activate ChR2. Note that a horizontally elongating axon is clearly labeled. The interrupted lines indicate the pial surface and the presumed layer 2/3 borders. Scale bar, 100 µm. **B**, Laminar localizations of the cells projecting horizontal axons which were used for the time-lapse study. Red dots indicate the locations of the cell bodies. Arrows indicate the presumed borders of layer 1, 2/3, 4, 5 and 6. **C,** Growth of the horizontal axon revealed by time-lapse study. The axon was followed every 10 min. Scale bar, 5 µm. **D,** Growth curve of the horizontal axon shown in **C**. **E,** Average growth of horizontal axons observed in cortical slice cultures from 3 to 10 DIV (n = 32).

A small number of upper layer cells were cotransfected with DsRed and ChR2-EYFP plasmids. ChR2-EYFP was used for optogenetic stimulation (475 nm), while DsRed was used to observe axonal growth under a different excitation light (560 nm) without activating ChR2. One day after electroporation, we detected strong and stable expression of DsRed/ChR2-EYFP in several cells. DsRed/ChR2-EYFP was highly expressed in the soma and axons, although the fluorescence intensity was slightly weaker in axons. After several days in culture, horizontally elongating axons were observed originating from pyramidal cells (Figure 1A), which is consistent with previous results [33], [34].

To examine their growth pattern, labeled axons were monitored every 10 min for several hours by time-lapse confocal microscopy with an excitation wavelength of 560 nm. Horizontally elongating axons from cells located at 100 to 300 µm (presumed layer 2/3) from the pial surface were selected for the observation (Figure 1B) [40]. We found that horizontally elongating axons grew continuously from 3 to 10 DIV, although the growth rates varied substantially among axons (Figure 1C, D, E). A quantitative analysis showed that the average growth rate was 29.9±1.7 µm/h (n = 32, Figure 1E), with 9.5 as minimal and 46.7 µm/h as maximal values.

### Light-induced Action Potentials

Next, we studied the effect of firing activity on axon growth by applying photostimulation at around one week in culture, when firing activity scarcely takes place [33].

**Figure 2:**
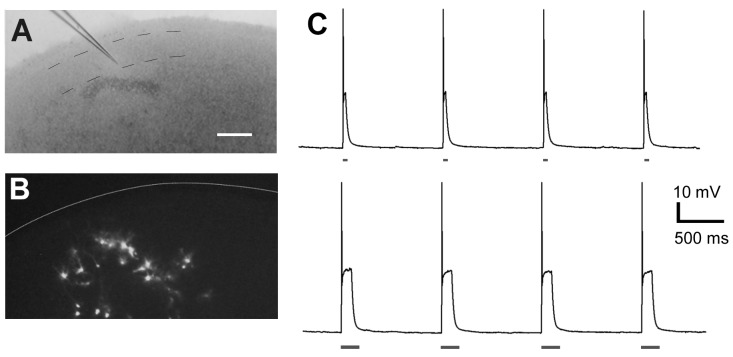
Electrophysiological responses of ChR2-expressing cortical neurons to photostimulation. **A** and **B**, ChR2-expressing cells among layer 2/3 neurons were subjected to patch-clamp recording at 4 DIV. Interrupted lines in **A** indicate the presumed layer 2/3 borders. The white line in **B** indicates the pial surface. Bar, 200 µm. **C,** Each flash with 475-nm wavelength light (50-msec duration in the upper trace and 200-msec duration in the lower trace) elicited depolarization and a single action potential.

To confirm that photostimulation produces firing activity in ChR2-expressing cells, whole-cell patch-clamp recording was performed (Figure 2). Photostimulation with different durations (50 or 200 msec) was applied to the ChR2-expressing cells. Each 50-msec flash of light elicited firing (Figure 2C, upper). A longer pulse duration (200 msec) also resulted in a single action potential (Figure 2C, lower).

No spike response was detectable in non-transfected neurons, indicating that the irradiance alone does not elicit a response, which is consistent with previous results [36], [44], [45]. Thus, we confirmed that photoactivation efficiently generated firing activity specifically in ChR2-expressing neurons.

### The Response of Horizontal Axons to High-frequency Photostimulation

Next, the behavior of horizontal axons was studied in response to photostimulation. The stimulation paradigm consisted of a 1-min photostimulation period with different frequencies (0.1, 1, 4 and 10 Hz).

**Figure 3:**
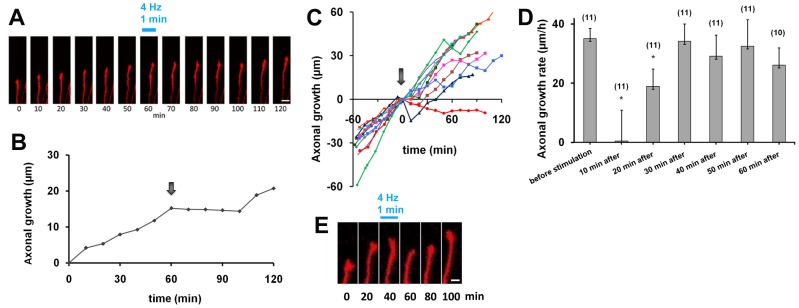
Horizontal axons exhibit pause behavior in response to high-frequency photostimulation directed at the soma of labeled neurons. **A,** A horizontal axon from a cell cotransfected with DsRed and ChR2-EYFP was observed. Photostimulation (4 Hz, 1 min) with 475-nm excitation was applied to the cell body after a 60-min observation of axon growth at 560-nm excitation. After the photostimulation at 475-nm excitation, the axon was further observed at 560-nm excitation. Scale bar, 5 µm. **B,** Growth of the horizontal axon shown in A. The arrow indicates the time of photostimulation. **C,** Growth of individual axons before and after photostimulation. Despite differences in growth rates, most axons display pause behavior after high-frequency photostimulation (arrow), n = 11. **D,** Axonal growth rates before and after high-frequency stimulation. After high-frequency photostimulation, axonal growth rate decreased significantly and this decline persisted for 20 min (Student’s *t*-test, *p<0.005). **E,** Axonal tip morphology before and after photostimulation. Scale bar, 2 µm.

First, high-frequency photostimulation was applied to the cell bodies which were the origins of labeled axons, as ChR2 was strongly expressed in the soma (Figure 1A). Before photostimulation, all observed axons were confirmed to elongate at constant growth rates for 30–60 min (35.1±3.5 µm/h, n = 11). We found that most of them transiently stopped growing after high-frequency photostimulation (4 and 10 Hz) (Figure 3A–C). A few axons showed obvious retraction. To quantify this axonal behavior, growth rates were calculated after photostimulation. The average growth rate was 0.47±9.9 µm/h (n = 11) at 10 min after photostimulation, significantly slower than the average before stimulation (Student’s *t*-test, p<0.005). Some axons started to grow again 20 min after photostimulation, but the average growth rate was still significantly lower (19.0±5.5 µm/h, p<0.05). Axonal growth rate was fully restored at 30 min after photostimulation (34.2±5.6 µm/h, Figure 3D).

Based on the above quantitative analysis, each axon’s behavior was categorized into pause and non-pause behavior. Because the 46% reduction in growth rate at 20 min after photostimulation (Figure 3D) was statistically significant, axons which exhibited more than a 50% decrease at this time point were defined as displaying pause behavior. Consequently, 9 of the 11 axons examined showed pause behavior. At 30 min after photostimulation, most of these (7 of the 9 axons) displayed fully restored growth rates, although the remaining two axons still exhibited significantly lower growth rates at this time point. All growth cones grew at normal rates by 50 min after photostimulation. We also observed the morphology of the growth cone. All axons exhibited typical growth cones with lamellipodia structures before photostimulation, but immediately after photostimulation the sizes of the lamellipodia decreased (Figure 3E).

**Figure 4:**
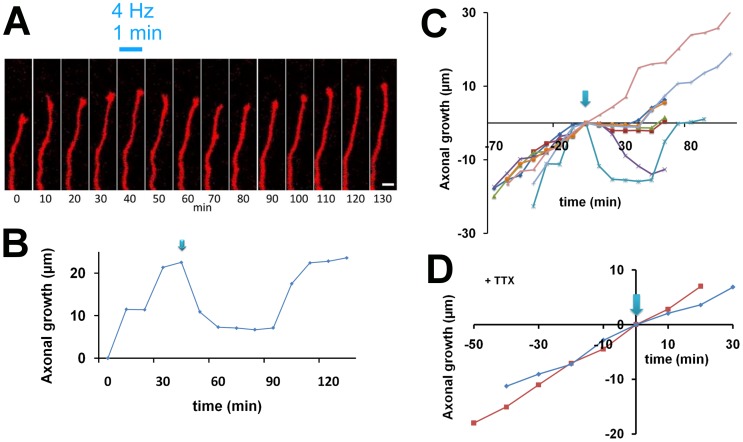
Horizontal axon growth before and after high-frequency stimulation to the axon. **A,** High-frequency photostimulation (4 Hz) elicited retraction of a representative growth cone. Scale bar, 5 µm. **B,** Growth of the horizontal axon shown in A. The arrow indicates the time of photostimulation. **C,** Growth of individual axons before and after photostimulation (arrow). **D,** Horizontal axon growth in the presence of TTX after high-frequency photostimulation. TTX (1 µM) was added to the culture medium 1 h prior to the observation. Photostimulation was applied at 4 Hz for 1 min to the cell body (n = 2).

A second time-lapse study was carried out after applying photostimulation directly to the axon, in order to explore whether action potentials generated in axons are required for the pause behavior (Figure 4A, B). Most of the observed axons exhibited slight retraction in response to photostimulation (Figure 4C), and quantitative analysis showed a marked decrease in axonal growth rate after photostimulation. The average growth rates (n = 8) at 10, 20, and 30 min after photostimulation were −8.5±9.0 µm/h, −4.9±3.9 µm/h, and −3.1±5.1 µm/h, respectively, displaying a significant reduction compared with that before stimulation (17.9±2.1 µm/h, Student’s *t*-test, p<0.005). Axonal growth rate was restored by 60 min (19.2±6.6 µm/h) after stimulation.

These axons were classified into two groups using the definition of pause behavior described above (i.e., more than 50% reduction at 20 min after stimulation). Seven axons out of eight exhibited pause behavior, while one axon was not obviously affected by the high-frequency photostimulation (Figure 4C). This result suggests that action potentials generated in axons at high frequencies also cause the pause behavior.

### Growth Cone Response in TTX-treated Culture

To further confirm that generation and propagation of action potentials are required for the pause behavior, the sodium channel blocker tetrodotoxin (TTX, 1 µM) was added to the culture medium one hour before the time-lapse experiment. Axons also grew normally, with growth cones, in blocker-containing medium. We found that in the presence of TTX, high-frequency photostimulation to the soma (4 Hz for 1 min) did not affect axonal growth rate (18.6±2.5 µm/h before stimulation, 21.8±6.8 µm/h at 10 min after stimulation and 14.8±3.3 µm/h at 20 min after stimulation, n = 2; Figure 4D). This result indicates that axons stop growing transiently due to firing activity elicited by photostimulation.

### The Influence of Low-frequency Photostimulation on Axon Growth

**Figure 5:**
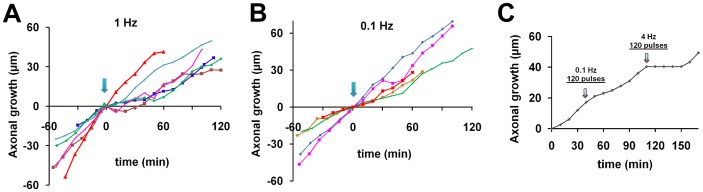
Axonal growth after low-frequency stimulation of the soma. **A, B,** Photostimulation at 1(**A**) or 0.1 Hz (**B**) for 1 min was applied (arrows) during the time-lapse study. **C,** A total of 120 pulses was applied to a single axon at low frequency (0.1 Hz) and subsequently at high frequency (4 Hz). Pause behavior was induced by high-frequency stimulation but not by low-frequency stimulation, even though the number of pulses was the same in both cases.

We investigated the influence of frequency of firing activity on axon growth. Photostimulation for 1 min at 1 Hz produced a weak effect on axonal growth (Figure 5A). The axons tested were classified as displaying pause and non-pause behavior, based on their growth rates before and after photostimulation (see above). Half of the axons did not show any pause (n = 3), whereas the others paused in their growth for 20 min (n = 3; Figure 5A).

Photostimulation for 1 min at 0.1 Hz did not result in any significant effect on the growth of any axon tested (Figure 5B). The growth rates were 23.5±7.2 µm/h, 34.7±9.6 µm/h and 38.4±5.3 µm/h at 10, 20, and 30 min after stimulation, respectively, and were not significantly different from that before stimulation (26.8±3.5 µm/h, n = 5; Figure 5B).

The total amount of firing activity may be critical for the pause behavior. To test this possibility, we increased the number of pulses while maintaining the frequency at 0.1 Hz. Even though the number of stimuli (120 pulses) was 20 times larger, axonal growth did not pause after the low-frequency stimulation. However, the same number of pulses applied subsequently to the same axon at high frequency (4 Hz, 120 pulses for 30 sec) elicited pause behavior for the next 40 min (Figure 5C). Therefore, the pause behavior depends on frequency rather than on the total amount of firing activity.

### Axonal Responses to Different Frequencies of Photostimulation

**Figure 6:**
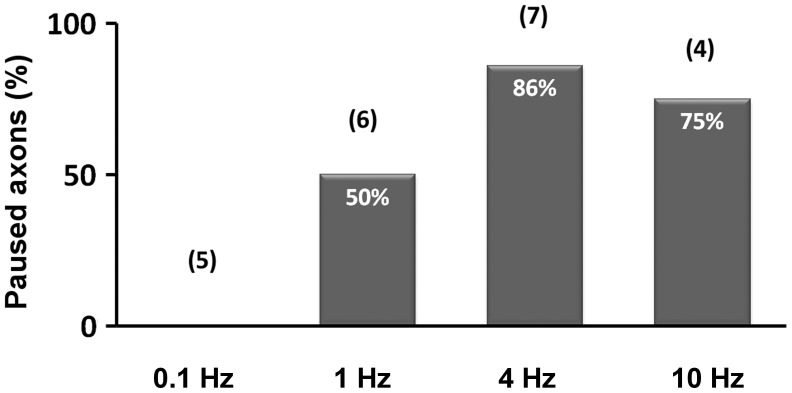
Occurrence of pause behavior after photostimulation with different frequencies. Histograms show the percentage of axons displaying pause behavior after photostimulation with different frequencies. The numbers in parentheses are the numbers of samples examined for each frequency.

The relationship between frequency of photostimulation and axonal response is shown in Figure 6. Based on the above analysis, high-frequency photostimulation was highly effective for the pause behavior (86% for 4 Hz, n = 7, 75% for 10 Hz, n = 4), intermediate stimulation was partly effective (50% for 1 Hz, n = 6), but low-frequency stimulation (0.1 Hz) had no effect on axonal growth (n = 5).

## Discussion

In the present study, we used optogenetic stimulation to investigate the influence of firing activity on cortical axon growth *in vitro*. The results demonstrate that horizontal axons grow at a constant rate after several days in culture, when spontaneous firing scarcely occurs, but exhibit pause behavior immediately after high-frequency firing is induced (see Figure 6). In the presence of TTX, photostimulation failed to elicit pause behavior. These results imply that high-frequency firing during development is involved in suppressing axon growth of layer 2/3 neurons and contributes to the formation of horizontal connections in the neocortex.

Combining time-lapse observation of a fluorescent protein and ChR2-expressing axons with optogenetic stimulation is also shown to be a powerful method for investigating activity-dependent processes in the brain. Using this approach, we demonstrate that firing activity contributes to the pause behavior and acts as a negative regulator for horizontal axon growth.

### Activity-dependence of Horizontal Axon Growth

As shown previously, spontaneous firing activity is almost absent in cortical slice culture during the first week *in vitro*, but begins to increase at around 10 DIV, reaching its peak at 14 DIV [33]. This temporal profile is compatible with the development of neuronal activity *in vivo* [46]–[48]. Taken together, the present findings suggest that horizontal axon growth is suppressed by increasing neuronal activity during cortical circuit formation.

The rapid response of the growth cone to high-frequency firing suggests that the pause behavior is cell-autonomous. Given that new branches tend to emerge during periods when axon growth has ceased, neuronal firing could strengthen the connectivity between cortical cells which are synchronously activated [43], [49]. As a consequence, only some of the horizontal axons originating from a layer 2/3 cell could form more extensive branches to make stronger connections with cortical cells in specific locations [24], [25], [32]. Consistent with this, our previous study has also shown that axonal branching of layer 2/3 cells is promoted by spontaneous activity and subsequent synaptic activity [33]. Overall, the present findings suggest that increasing spike activity during postnatal stages contributes to the establishment of horizontal connections by inhibiting axon growth and promoting branch formation.

However, it should be noted that an intrinsic mechanism also contributes to axon branching of layer 2/3 cells, because some aspects of their branching pattern are highly preserved irrespective of environmental conditions [49], [50].

### The Efficiency of Neuronal Activity

Even after high-frequency stimulation (4 Hz and 10 Hz), not all of the tested axons showed pause behavior. When intermediate-frequency stimulation (1 Hz) was applied, only half of the examined axons paused growing. As there are subclasses among upper-layer pyramidal cells [23], [51], distinct populations may respond differentially to firing activity. Another possibility is that the history of neuronal activity may influence axonal responses, as a growth cone that has experienced electrical stimulation-induced collapse becomes insensitive to a further stimulation [52].

### Possible Mechanisms of Growth Cone Pausing in Response to Neuronal Activity

Previous studies have shown that axon growth is arrested in peripheral and invertebrate neurons when the cells are electrically stimulated [53], [54]. Moreover, it has been demonstrated that the suppression of growth cone motility is attributable to a rise in Ca^2+^ concentration in the growth cones in the PNS [55]–[57]. Similarly, Ca^2+^ elevation may be essential for the pause behavior of horizontal axons in the cortex.

Is gene expression involved in the observed pause behavior? In the classical collapse assay, addition of the repulsive guidance molecule Sema-3A is known to induce immediate growth cone collapse, which lasts for 30–60 min [58]. The same phenomenon was observed even in axons that were isolated from the soma. This indicates that the effect is independent of gene expression. As axonal responses in the present study resemble Sema-3A-induced growth cone collapse in temporal terms, it is likely that the pause behavior is also independent of nuclear gene expression. However, local translation in axonal tips may be partly responsible for the pause behavior of cortical axons [59], [60].

It has also been shown that electrical activity alters growth cone responsiveness to environmental factors. For instance, light electrical stimulation of *Xenopus* motoneurons (10 action potentials at 2 Hz), which by itself does not induce any change in axonal outgrowth, is able to lower the sensitivity of growth cones to attractive guidance cues or to convert repulsive cues into attractive ones [61]. Thus, growth cone responses may be affected differentially by the strength of electrical activity. *In vitro* experiments in developing rat neocortical slices demonstrate the presence of spatiotemporally organized patterns of spontaneous cortical activity [62], [63]. It is likely that such unique firing patterns can also influence axon behavior [64], [65].

In summary, various intrinsic and extrinsic neuronal events including firing and synaptic activity probably contribute to the decision of an axon to advance, retract, pause, or turn. The present study indicates that firing activity is also an influential factor for axonal development in the mammalian neocortex, which is crucial for the formation of its columnar structure.
